# MaMADS1–MaNAC083 transcriptional regulatory cascade regulates ethylene biosynthesis during banana fruit ripening

**DOI:** 10.1093/hr/uhad177

**Published:** 2023-09-08

**Authors:** Wei Wei, Ying-ying Yang, Chao-jie Wu, Jian-fei Kuang, Jian-ye Chen, Wang-jin Lu, Wei Shan

**Affiliations:** Guangdong Provincial Key Laboratory of Postharvest Science of Fruits and Vegetables/Engineering Research Center of Southern Horticultural Products Preservation, Ministry of Education, College of Horticulture, South China Agricultural University, Guangzhou 510642, China; Guangdong Provincial Key Laboratory of Postharvest Science of Fruits and Vegetables/Engineering Research Center of Southern Horticultural Products Preservation, Ministry of Education, College of Horticulture, South China Agricultural University, Guangzhou 510642, China; Guangdong Provincial Key Laboratory of Postharvest Science of Fruits and Vegetables/Engineering Research Center of Southern Horticultural Products Preservation, Ministry of Education, College of Horticulture, South China Agricultural University, Guangzhou 510642, China; Guangdong Provincial Key Laboratory of Postharvest Science of Fruits and Vegetables/Engineering Research Center of Southern Horticultural Products Preservation, Ministry of Education, College of Horticulture, South China Agricultural University, Guangzhou 510642, China; Guangdong Provincial Key Laboratory of Postharvest Science of Fruits and Vegetables/Engineering Research Center of Southern Horticultural Products Preservation, Ministry of Education, College of Horticulture, South China Agricultural University, Guangzhou 510642, China; Guangdong Provincial Key Laboratory of Postharvest Science of Fruits and Vegetables/Engineering Research Center of Southern Horticultural Products Preservation, Ministry of Education, College of Horticulture, South China Agricultural University, Guangzhou 510642, China; Guangdong Provincial Key Laboratory of Postharvest Science of Fruits and Vegetables/Engineering Research Center of Southern Horticultural Products Preservation, Ministry of Education, College of Horticulture, South China Agricultural University, Guangzhou 510642, China

## Abstract

The hormone ethylene is crucial in the regulation of ripening in climacteric fruit, such as bananas. The transcriptional regulation of ethylene biosynthesis throughout banana fruit ripening has received much study, but the cascaded transcriptional machinery of upstream transcriptional regulators implicated in the ethylene biosynthesis pathway is still poorly understood. Here we report that ethylene biosynthesis genes, including *MaACS1*, *MaACO1*, *MaACO4*, *MaACO5*, and *MaACO8*, were upregulated in ripening bananas. NAC (NAM, ATAF, CUC) transcription factor, MaNAC083, a ripening and ethylene-inhibited gene, was discovered as a potential binding protein to the *MaACS1* promoter by yeast one-hybrid screening. Further *in vitro* and *in vivo* experiments indicated that MaNAC083 bound directly to promoters of the five ethylene biosynthesis genes, thereby transcriptionally repressing their expression, which was further verified by transient overexpression experiments, where ethylene production was inhibited through MaNAC083-modulated transcriptional repression of ethylene biosynthesis genes in banana fruits. Strikingly, MaMADS1, a ripening-induced MADS (MCM1, AGAMOUS, DEFICIENS, SRF4) transcription factor, was found to directly repress the expression of *MaNAC083*, inhibiting *trans*-repression of MaNAC083 to ethylene biosynthesis genes, thereby attenuating MaNAC083-repressed ethylene production in bananas. These findings collectively illustrated the mechanistic basis of a MaMADS1–MaNAC083–*MaACS1/MaACO*s regulatory cascade controlling ethylene biosynthesis during banana fruit ripening. These findings increase our knowledge of the transcriptional regulatory mechanisms of ethylene biosynthesis at the transcriptional level and are expected to help develop molecular approaches to control ripening and improve fruit storability.

## Introduction

Ethylene, a versatile phytohormone, modulates numerous physiological processes, spanning seed germination to organ senescence [1]. Due to its critical involvement in regulating agronomically valuable traits in plants, particularly fruit ripening and quality formation, ethylene production has been a topic of intense study [2]. The ethylene biosynthetic pathway includes two enzyme steps. *S*-Adenosyl-l-methionine (SAM) is converted to 1-aminocyclopropane-1-carboxylic acid (ACC) via catalysis by ACC synthase (ACS) [3]. The second step, converting ACC to ethylene, is catalyzed through ACC oxidase (ACO) [4]. Thus, ethylene production is controlled by ACS and ACO, the rate-limiting enzymes for ethylene biosynthesis. Multigene families that encode the enzymes ACS and ACO are a longstanding area of interest in fruit ripening research [5].

Gene expression regulation requires precise transcriptional programs, involving binding of transcription factors (TFs) to *cis*-acting regions of gene promoter DNA [6]. With the advent of deep research on tomato non-ripening mutants *rin* and *nor*, researchers have steadily been interested in the central TFs' role in regulating fruit ripening. Members of the MADS and NAC families of TFs are encoded by the *nor* and *rin* genes, respectively [7]. RIN and NOR were found to regulate fruit ripening by acting upstream of the ethylene pathway, as evaluated by the phenotypic characterization of mutants [8]. Throughout tomato fruit ripening, RIN is the first transcription factor discovered to control ethylene biosynthetic gene expression, including *SlACS2* and *SlACO4* [9, 10]. NOR was also found to favorably control ethylene biosynthesis through direct induction of *SlACS2* and *SlACS4* [11]. Regulation of the ACS and ACO genes throughout fruit ripening (apples, plums, bananas, and melons) is also believed to be controlled by other TFs, including ERF, MYB, MYC, and ABF [12–15].

**Figure 1:**
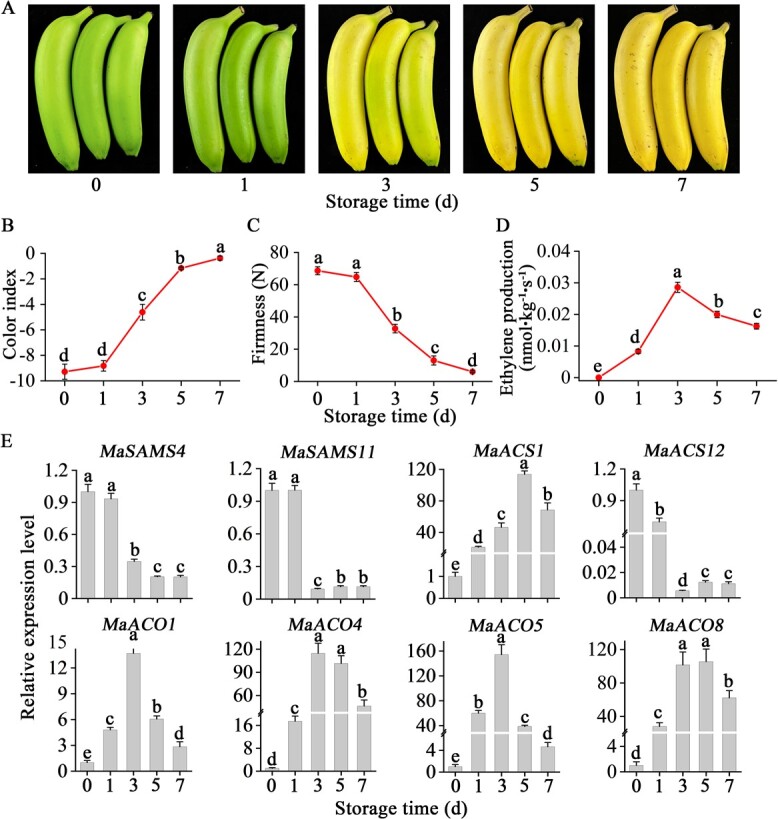
Ethylene production and ethylene biosynthesis genes expression in banana fruit during ethylene-induced ripening. (A) Ethylene-induced banana ripening phenotype alteration. (B–D) CI changes (B), fruit firmness (C), and ethylene generation (D) in fruit during ripening. (E) Banana fruit ripening expression of *MaSAMS4*, *MaSAMS11*, *MaACS1*, *MaACS12*, *MaACO1*, *MaACO4*, *MaACO5*, and *MaACO8*. The expression level is represented as a ratio to day 0 (set at 1). Data are represented as means ± standard error from *n* = 6 (B-–D) and *n* = 3 (E) replicates, and different letters denote statistical variation (one-way ANOVA, *P* < .05).

The banana (*Musa acuminata*) is the most widespread type of fresh fruit in the world, produced mainly in developing countries due to the special climatic conditions needed to grow them, and are a multimillion-dollar export product consumed primarily in wealthier nations [16]. Currently, commercialized bananas are dominated by Cavendish cultivars with an AAA triploid genome [17]. Bananas are typically collected in a green, mature phase and shipped to wholesale markets, where they are artificially ripened to a golden yellow fruit with good edible quality by application of ethylene [18]. Hence, the regulating mechanisms of ethylene production during postharvest banana fruit ripening have received considerable study. During postharvest ripening, ACS and ACO are induced and modulate ethylene biosynthesis in banana fruit [19, 20]. *MaACS1* (also *MaACS7* [21], Ma04_t35640) and *MaACO1* (also *MaACO8*, [21] Ma07_g19730) are widely recognized as key ethylene biosynthesis genes involved in banana ripening [22]. Various ethylene signaling and biosynthesis-related gene groups have been further investigated in banana fruit utilizing transcriptome and genome profiling [21, 23]. Furthermore, there is a significant emphasis on the transcriptional regulation of the genes responsible for ethylene biosynthesis in bananas. Multiple transcription factors have been identified to have critical functions in banana fruit ripening, including ERF [13], bHLH [18], NAC [24], EIL [25], and MYB [26]. MaNAC029 and MaERF9 function as positive regulators, while MaERF11 is a negative transcriptional regulator of the ethylene biosynthetic pathway [13, 20, 24]. However, the relationship among these upstream transcription factors of the ethylene biosynthesis pathway, especially their cascaded transcription mechanism, remains unclear.

Therefore, this study aims to analyze the cascaded transcriptional machinery of ethylene biosynthesis during banana fruit ripening. The findings of this research will offer novel transcriptional insights into fruit ripening and provide clues for improving banana storage and for breeding high-quality fruits.

**Figure 2:**
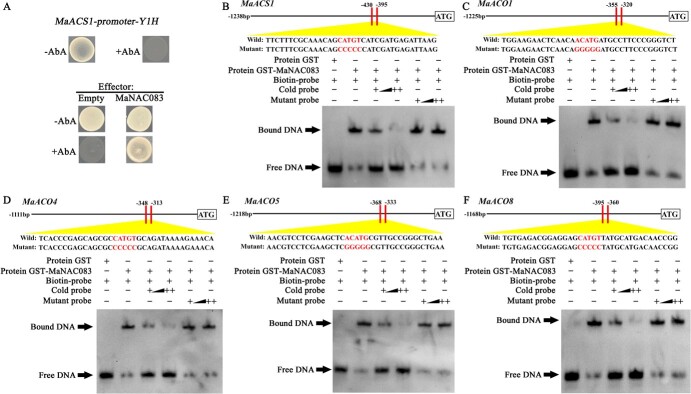
MaNAC083 bound to promoters of *MaACS1*, *MaACO1*, *MaACO4*, *MaACO5*, and *MaACO8*. (A) Y1H study of MaNAC083's effect on the *MaACS1* promoter. (Top) The *MaACS1* promoter was not expressed in yeast developed on SD media without Leu and with 500 ng mL^−1^ AbA. (Bottom) A yeast growth assay was conducted after introducing plasmids containing cassettes that expressed either MaNAC083 effector or empty (pGADT7; negative control) to Y1H reporter strains. The interaction between them was evaluated based on their capacity to grow when transformed yeast was cultivated in SD medium without Leu under the influence of AbA. (B) MaNAC083 binding to *MaACS1*, *MaACO1*, *MaACO4*, *MaACO5*, and *MaACO8* promoters via EMSA. The wild type and mutant probe sequences for ethylene biosynthesis gene promoters are shown at the top. Purified GST protein (negative control) or recombinant GST-MaNAC083 protein was subjected to an incubation process along with probes. The resulting DNA–protein complexes were separated on native polyacrylamide gels. Triangles show competing quantities of unlabeled wild-type or mutated probes.

## Results

### Identification of key genes for ethylene biosynthesis related to banana fruit ripening

RNA-seq of banana fruits in different stages was conducted to detect potential ethylene biosynthesis genes associated with fruit ripening. This transcriptome analysis revealed nine ethylene biosynthesis genes, of which one *ACS* gene and four *ACO* genes were induced by ripening, and two *SAM synthetase* (*SAMS*) genes and one *ACS* gene were repressed by ripening (Supplementary Data Fig. S1 and Supplementary Data Table S1). These ripening-related genes, including *MaSAMS4*, *MaSAMS11*, *MaACS1*, *MaACS12*, *MaACO1*, *MaACO4*, *MaACO5*, and *MaACO8*, were subsequently selected, and their expression pattern during the whole ripening process was measured by qRT–PCR. After the initiation of ripening with exogenous ethylene treatment, a color change was initiated in the banana peel from green to yellow on the third day and the peel was completely yellow on the fifth day (Fig. 1A), which was also indicated by a sharp rise in the color index (CI) from −9.28 at day 0 to −0.37 on the seventh day (Fig. 1B). Pulp firmness reduced progressively after 3 days of storage and ultimately dropped to a minimum level of 6.02 N on the seventh day (Fig. 1C). The endogenous generation of ethylene in banana fruits elevated significantly after 1 storage day, reaching a maximum on day 3 and then declining (Fig. 1D). In parallel, the expression of five ethylene biosynthesis genes, *MaACS1*, *MaACO1*, *MaACO4*, *MaACO5*, and *MaACO8*, was initially elevated, peaked on the third or fifth day, and subsequently marginally declined during ripening, consistent with the ethylene production (Fig. 1E). However, the expression of *MaSAMS4*, *MaSAMS11*, and *MaACS12* showed downward trends during the postharvest ripening process. These results suggested that *MaACS1*, *MaACO1*, *MaACO4*, *MaACO5*, and *MaACO8* are good candidates associated with ethylene biosynthesis during banana ripening.

**Figure 3:**
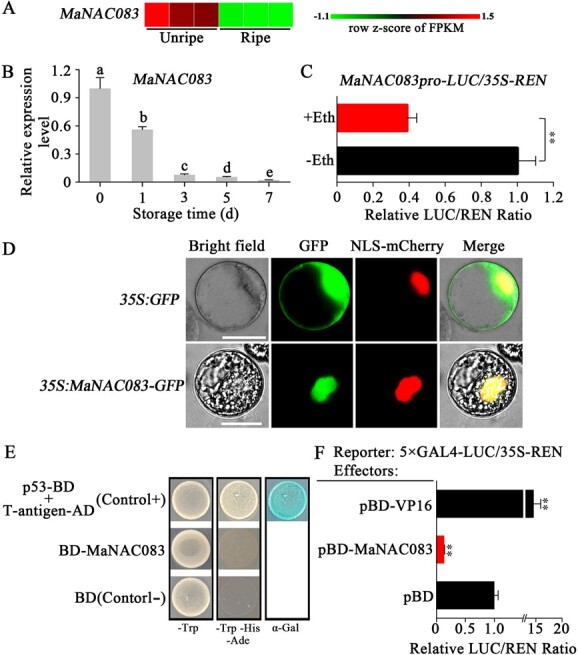
MaNAC083 molecular characteristics. (A) Heat map of gene expression of *MaNAC083* in transcriptome analysis of unripe and ripe fruit. (B) *MaNAC083* expression in ripening banana fruit. Data are means ± standard error from three replicates and different letters denote statistical variation (one-way ANOVA, *P* < .05). (C) *MaNAC083* promoter activity responding to ethylene. (D) MaNAC083 localization in tobacco BY-2 protoplasts. NLS-mCherry was utilized as a nuclear marker. Scale bars, 25 μm. (E) MaNAC083 transcriptional stimulation in yeast cells. The full MaNAC083 length was merged with the GAL4 DNA-binding domain. Two controls were employed: the pGBKT7 vector (BD) served as a negative control, while p53-BD + T-antigen-AD functioned as a positive one. (F) MaNAC083 transcriptional activity in tobacco leaves. Using VP16 (an extremely strong transcriptional activator) as a positive control, we standardized the LUC/REN ratio of the empty pBD vector (negative control) to 1. Error bars in (C) and (F) denote the standard error from six replicates. Student's *t*-test, ^**^P < .01.

### MaNAC083 directly targets ethylene biosynthesis genes

To understand the regulatory techniques that influence the development of ethylene biosynthetic genes, we conducted a screening process utilizing yeast one-hybrid (Y1H) libraries in search of proteins interacting with their respective promoters. Due to the inability of aureobasidin A (AbA) to inhibit promoter activities of *MaACO1*, *MaACO4*, *MaACO5*, and *MaACO8*, the banana fruit cDNA library was screened using the *MaACS1* promoter as bait. After screening, a fragment of NAC gene cDNA (*Ma10_g12120*) was detected. Previous studies showed that Ma10_g12120 has the closest evolutionary relationship with *Arabidopsis* ANAC083 [27]; hence, it was designated MaNAC083. The Y1H assay validated the interaction between MaNAC083 and the *MaACS1* promoter by employing *MaNAC083* full-length coding DNA sequences as prey. The *MaACS1* promoter did not exhibit basal activity in yeast with AbA, while yeast cells expressing *MaNAC083* triggered the AbA resistance gene expression controlled by the *MaACS1* promoter and developed well on media containing AbA, showing that MaNAC083 protein physically interacts with the *MaACS1* promoter (Fig. 2A).

Promoter analysis of *MaACS1*, *MaACO1*, *MaACO4*, *MaACO5*, and *MaACO8* showed the presence of the NAC recognition sequence (NACRS) in their promoters (Supplementary Data Fig. S2). *In vitro*, an electrophoretic mobility shift assay (EMSA) was utilized to determine if MaNAC083 can interact with all promoters directly. The purified recombinant MaNAC083 protein (Supplementary Data Fig. S3) bound to NACRS-containing fragments from these five promoters, resulting in mobility changes that were reversed by unlabeled probes with the same sequence but without altered competitors (Fig. 2B–F).

These data illustrate that MaNAC083 directly targets *MaACS1*, *MaACO1*, *MaACO4*, *MaACO5*, and *MaACO8* promoters via the NACRS.

### Molecular characterization of MaNAC083

RNA-seq data analysis revealed that *MaNAC083* mRNA levels were lower in ripe banana fruit than in unripe fruit (Fig. 3A). The expression pattern during the ethylene-induced ripening process was further measured by RT–qPCR to investigate the potential correlation between *MaNAC083* and fruit ripening. The *MaNAC083* gene transcripts obviously decreased during ripening (Fig. 3B). Furthermore, ethylene decreased the activity of the *MaNAC083* promoter, as illustrated by a transient expression assay based on the dual-luciferase reporter (DLR) system (Fig. 3C). To investigate the subcellular localization, GFP tagged with MaNAC083 was transiently produced in tobacco BY2 protoplasts. Then, the nucleus-targeted mCherry (NLS-mCherry) was co-expressed as a control to monitor the nucleus. The result demonstrated that GFP control fluorescence could be seen in the cytoplasm and nucleus. The green and red fluorescent signals of MaNAC083-GFP and NLS-mCherry showed nuclear localization (Fig. 3D). MaNAC083's transcriptional ability was examined utilizing a GAL4-responsive yeast reporter system. Full-length MaNAC083 exhibited self-transcriptional activation activity among yeast cells (Fig. 3E). Utilizing the DLR system, MaNAC083's transcriptional ability was further evaluated in *Nicotiana benthamiana* leaves by coupling the LUC reporter with a TATA box and five GAL4 DNA-binding components. VP16, a potent transcriptional stimulator, served as the positive control (Fig. 3F). MaNAC083 considerably decreased the LUC/REN ratio compared with an empty BD vector. These findings indicated that MaNAC083 could be a transcriptional repressor that mediates banana ripening.

### MaNAC083 represses the expression of ethylene biosynthesis genes and inhibits ethylene production

**Figure 4:**
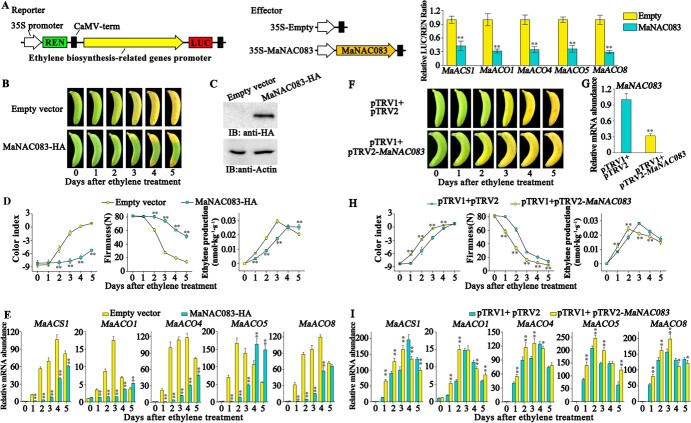
MaNAC083 inhibits ethylene biosynthesis gene expression and ethylene production. (A) MaNAC083 represses the activity of *MaACS1*, *MaACO1*, *MaACO4*, *MaACO5*, and *MaACO8* promoters in a DLR assay in tobacco leaves. Schematic representation of reporter and effector constructs is illustrated in the left panel. LUC/REN ratio of an empty vector with ethylene biosynthetic gene promoters was utilized as a calibrator (set to 1). (B–E) Transient overexpression of *MaNAC083* in banana fruit. (B) During ripening, *MaNAC083* and empty vector were transiently overexpressed in banana fruit. (C) Western blot showing MaNAC083 protein level transiently *MaNAC083*-overexpressing and empty vector in bananas. Protein detection employed fruit pulp tissues from the injection region . Actin was detected as the loading control. (D) CI, firmness, and ethylene production of *MaNAC083*-overexpressing and control banana fruit throughout the ripening process. (E) Relative expression of *MaACS1*, *MaACO1*, *MaACO4*, *MaACO5*, and *MaACO8* in MaNAC083-overexpressing and control banana fruit. (F–I) Transient silencing of *MaNAC083* in banana fruit. (F) Appearance of banana fruit infiltrated with empty vector (control) and pTRV2-*MaNAC083*. (G) RT–qPCR showing *MaNAC083* mRNA level in infiltrated banana fruit. (H) CI changes, firmness, and ethylene production in banana fruit as shown in (F). (I) Relative expression of *MaACS1*, *MaACO1*, *MaACO4*, *MaACO5*, and *MaACO8* in banana fruit as shown in (F). Error bars in (A, D, H), and (E, G, I) denote standard error from six replicates and triplets, respectively. Student's *t*-test, ^**^P < .01.

Since MaNAC083 revealed transcriptional repression activity and was directly bound to ethylene biosynthesis gene promoters (Figs 2 and3E–F), a transient DLR assay was conducted to assess if MaNAC083 represses ethylene biosynthesis gene transcription. The *MaACS1*, *MaACO1*, *MaACO4*, *MaACO5*, and *MaACO8* promoter activities were inhibited after MaNAC083 co-transfection when compared with co-transfection with an empty vector. A lower LUC/REN ratio was reported, showing that MaNAC083 could repress the ethylene biosynthesis gene promoter (Fig. 4A).

To verify the MaNAC083-mediated transcriptional regulation of ethylene biosynthesis genes in bananas, we performed transient overexpression and silencing of *MaNAC083* in banana fruit. Banana fruit with transiently overexpressed *MaNAC083* showed slowed fruit ripening, and *MaNAC083-*overexpressing fruit revealed yellowing reduction compared with controls (Fig. 4B and C). Also, *MaNAC083*-overexpressing fruit exhibited reduced CI and a higher firmness than control fruit (Fig. 4D). Furthermore, *MaNAC083* overexpression significantly inhibited ethylene generation compared with the control (Fig. 4D). In parallel, the expressions of *MaACS1*, *MaACO1*, *MaACO4*, *MaACO5*, and *MaACO8* were downregulated in the overexpressing fruits during ripening (Fig. 4E). In contrast, suppression of endogenous *MaNAC083* expression through virus-induced gene silencing (VIGS) hastened fruit ripening and resulted in a faster yellowing phenotype (Figs. 4F–G). Concomitantly, higher CI and lower firmness of the fruit were found in *MaNAC083*-silenced fruit (Fig. 4H). More importantly, suppression of *MaNAC083* expression significantly accelerated the rise in ethylene production relative to control fruit (Fig. 4H). In parallel, *MaACS1*, *MaACO1*, *MaACO4*, *MaACO5*, and *MaACO8* in *MaNAC083*-silenced fruits were significantly upregulated compared with the control fruit during the ripening progress (Fig. 4I).

These results demonstrated that MaNAC083 inhibits ethylene biosynthesis in banana ripening by blocking *MaACS1*, *MaACO1*, *MaACO4*, *MaACO5*, and *MaACO8* gene activities.

### MaMADS1 directly binds to the *MaNAC083* promoter

**Figure 5:**
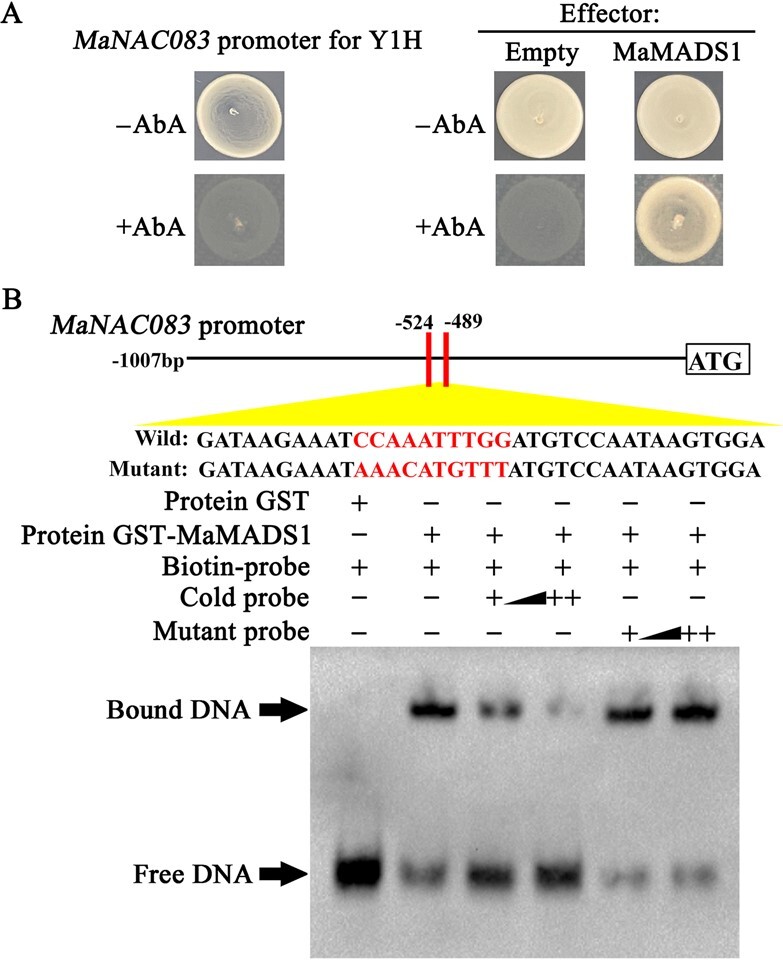
MaMADS1 directly binds to the *MaNAC083* promoter. (A) Y1H study of MaMADS1's interaction with the *MaNAC083* promoter. (Left) The *MaNAC083* promoter showed no basal expression in yeast grown on Leu-deficient SD media supplemented with 500 ng mL^−1^ AbA. (Right) After transforming plasmids containing cassettes that constitutively express the MaMADS1 effector or negative control (pGADT7), a yeast growth assay was performed on Y1H reporter strains. The interaction was evaluated by yeast growth after transformation on SD media without Leu in AbA's presence. (B) MaMADS1 binding to *MaNAC083* promoter in an EMSA assay. The probe sequences for ethylene biosynthetic gene promoters are displayed at the top. DNA–protein complexes from probes exposed to pure GST (negative control) or recombinant GST-MaMADS1 protein were resolved on native polyacrylamide gels. Triangles show competing quantities of unlabeled wild-type or mutated probes.

To assess *MaNAC083* upstream regulators, a Y1H screen was done utilizing the *MaNAC083* promoter as bait. Therefore, a cDNA fragment encoding the MADS transcription factor MaMADS1 peptide was obtained [28]. Utilizing full-length MaMADS1 as prey in one-to-one Y1H assays, the relationship between the MaMADS1 and MaNAC083 promoters was further verified. Yeast cells developed well on AbA-containing media when co-expressing MaMADS1, which promoted the AbA resistance gene expression regulated by the MaNAC083 promoter (Fig. 5A).

It is generally accepted that MADS TFs tend to bind to their target promoters' CArG boxes [29]. Sequence analyses have considered the CArG box in the *MaNAC083* promoter (Supplementary Data Fig. S4). Subsequently, we performed an EMSA, which showed that the purified MaMADS1 (Supplementary Data Fig. S5) directly bound to the CArG box with a fragment derived from the *MaNAC083* promoter and resulted in obvious mobility shifts (Fig. 5B). Furthermore, the introduction of unlabeled probes possessing identical sequences resulted in the disappearance of displaced bands. In contrast, mutated competitors had no similar effect.

Together, these studies demonstrate that MaMADS1 specifically targets the *MaNAC083* promoter.

### MaMADS1 promotes ethylene biosynthesis through transcriptional repression of *MaNAC083*

**Figure 6:**
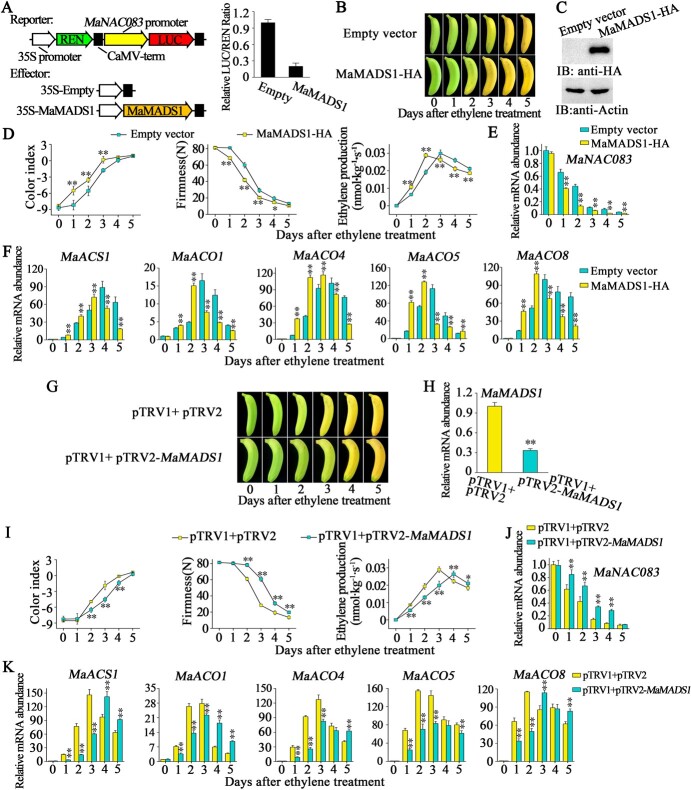
MaMADS1 inhibits *MaNAC083* expression and promotes ethylene biosynthesis. (A) MaMADS1 represses *MaNAC083* promoter activity in a DLR assay in tobacco leaves. The left panel shows reporter and effector constructs schematically. For the empty vector with the ethylene biosynthesis gene promoter reporter, the LUC/REN ratio was set as 1. (B–F) Transient overexpression of *MaMADS1* in banana fruit. (B) Banana fruit ripening transiently overexpresses *MaMADS1* and empty vector. (C) Western blot showing MaMADS1 protein level in bananas transiently overexpressing empty vector and *MaMADS1*. Protein recognition used fruit pulp tissues from the injection area, with actin as a loading control. (D) CI, firmness, and ethylene production during the ripening process in *MaMADS1*-overexpressing and control banana fruit. (E) Relative expression of *MaNAC083* in *MaMADS1*-overexpressing and control banana fruit. (F) *MaACS1*, *MaACO1*, *MaACO4*, *MaACO5*, and *MaACO8* gene expression in *MaMADS1*-overexpressing and control bananas. (G–K) Transient silencing of *MaMADS1* in banana fruit. (G) Appearance of banana fruit infiltrated with empty vector (control) and pTRV2-*MaMADS1*. (H) RT–qPCR showing *MaMADS1* mRNA level in infiltrated banana fruit. (I) Changes in CI, firmness, and ethylene production in banana fruit as shown in (G). (J) Relative expression of *MaNAC083* in banana fruit as shown in (G). (K) Expression of *MaACS1*, *MaACO1*, *MaACO4*, *MaACO5*, and *MaACO8* in banana fruit as shown in (G). Error bars in (A, D, G, I) and (E, F, J, K) denote the standard error from six replicates and triplets, respectively. Student's *t*-test, ^**^P < .01.

The expression of the *MaMADS1* gene was ripening-induced, and its protein possessed transcriptional repression activity (Supplementary Data Fig. S6), so we postulated that MaMADS1 could transcriptionally repress the expression of *MaNAC083*. To verify this speculation, a transient DLR-based *trans*-activation was conducted. The promoter activity of *MaNAC083* was greatly decreased in the presence of MaMADS1, with a considerably lower LUC/REN ratio than the control (Fig. 6A).

To assess the role of MaMADS1 in regulating MaNAC083 and its downstream targets, banana fruit exhibited transient overexpression and silencing. Rapid ripening of banana fruit was induced by transient overexpression of *MaMADS1*, as *MaMADS1-*overexpressing fruits showed accelerated yellowing compared with the control fruits (Fig. 6B and C). Furthermore, *MaMADS1* overexpression significantly accelerated the increase in CI and ethylene production and the decline in fruit firmness compared with the control (Fig. 6D). Noticeably, the *MaNAC083* transcript level was obviously inhibited in *MaMADS1*-overexpressing fruits compared with the control (Fig. 6E). In contrast, the expression of *MaACS1*, *MaACO1*, *MaACO4*, *MaACO5*, and *MaACO8* was dramatically upregulated in *MaMADS1-*overexpressing fruit during ripening (Fig. 6F). However, suppression of *MaMADS1* expression delayed fruit ripening, as the *MaMADS1*-silencing fruit showed a less yellowing peel compared with control fruit (Fig. 6G–H). In addition, silencing of *MaMADS1* in banana fruit significantly retarded the rise in CI and ethylene production and the decline in fruit firmness during ripening (Fig. 6I). Furthermore, *MaNAC083* was upregulated but *MaACS1*, *MaACO1*, *MaACO4*, *MaACO5*, and *MaACO8* were downregulated in *MaMADS1*-silenced fruit (Fig. 6J–K).

These findings confirmed that MaMADS1 inhibits the expression of *MaNAC083*, which then attenuates MaNAC083-mediated repression of ethylene biosynthesis genes and thus promotes ethylene biosynthesis in banana fruit.

## Discussion

Ethylene is a vital hormone for modulating climacteric fruit ripening, including bananas [25, 30]. On the one hand, ethylene triggers banana ripening to promote fruit quality formation, while on the other hand, it accelerates fruit ripening and senescence to decrease shelf life [31, 32]. Hence, investigation of the regulatory mechanism of ethylene production throughout banana ripening is crucial for fruit quality improvement and shelf life extension. Fruit ripening is a finely controlled process that includes several TFs [33]. Various research has identified the role of multiple TFs in banana ripening-associated transcriptional mechanisms [34, 35], but the upstream transcriptional regulator cascade involved in the ethylene biosynthesis pathway remains unclear. Here we discuss and identify the molecular mechanism for the regulation of ethylene biosynthesis mediated by the MaMADS1–MaNAC083 transcription factor cascade through banana ripening.

The induction of *ACS* and *ACO* genes is crucial in ethylene biosynthesis regulation during climacteric fruit ripening [3, 5]. Earlier studies found that the expression of *MaACS1* (also *MaACS7* [21], Ma04_t35640) and *MaACO1* (also *MaACO8* [21], Ma07_g19730) increases significantly at banana ripening onset, consistent with ethylene production [22, 23]. Genome-wide transcript profiling further showed that, apart from *MaACS1* and *MaACO1*, other *ACO* genes, such as *MaACO5* (also *MaACO2*, Ma05_g09360) and *MaACO8* (also *MaACO11*, Ma10_g16100) exhibit high expression levels during postharvest banana ripening [21]. Consistently, our present study demonstrated that the expression patterns of *MaACS1*, *MaACO1*, *MaACO4*, *MaACO5*, and *MaACO8* generally align with ethylene production during banana ripening (Fig. 1). Together, these findings confirm the pattern of ethylene biosynthesis gene expression during banana ripening and serve as a foundation for future investigations into the regulatory network governing banana ripening.

NAC transcription factors, an extensive group of plant-specific transcription factors, serve crucial functions in plant growth, development, and stress responses [36, 37]. The non-ripening tomato mutant *nor* was identified and NAC TFs' role in fruit ripening was investigated [7, 38]. The *nor* mutation significantly inhibits ethylene production and fruit quality formation [39, 40]. Additional molecular investigation reveals that NAC-NOR binds directly to the promoter and enhances the expression of the ethylene biosynthesis gene *SlACS2* [11]. Furthermore, SlNAC1 and SlNOR-like1 have been found to directly regulate both ACS and ACO gene expressions in tomato ripening [41, 42]. Several additional members of the NAC family in other fruit, such as kiwifruit AdNAC2/3 and banana MaNAC029, have also been implicated in fruit ripening by controlling ethylene production genes [43]. However, these NAC TFs are all positive transcriptional regulators that function upstream of the ethylene biosynthesis pathway. NAC-mediated negative transcriptional regulation of ethylene biosynthesis is unclear. The banana genome encodes 167 NACs [22], among which MaNAC083 is a putative ortholog of *Arabidopsis* negative regulator of senescence ANAC083 (VNI2) [44]. However, to date no biological function has been identified for MaNAC083. This study found that MaNAC083 is a transcriptional repressor that directly binds to the promoters of the *MaACS1*, *MaACO1*, *MaACO4*, *MaACO5*, and *MaACO8* genes and represses their expression, which in turn inhibits ethylene biosynthesis and banana ripening (Figs 2–4). These findings illustrate the mechanism of the negative regulatory function of MaNAC083 in ethylene biosynthesis, enhancing our information on how NAC TFs regulate fruit ripening.

Fruit ripening is a coordinated transcriptional regulatory process that is mediated by a large count of transcription factors [33]. Until now, several TFs belonging to different families have been implicated in banana ripening through direct regulation of ripening-related structural genes. For example, MaERF9, MaMYB4, and MabZIP21 have been shown to implicate fruit softening and ethylene synthesis by directly controlling the transcription of ethylene biosynthesis and cell wall modifying genes [13, 32, 45]. MabHLH6 and MaMYB3 directly target starch degradation-related genes to positively and inversely modulate starch degradation throughout the ripening process, respectively [18, 34]. Furthermore, MaMYB60 and MaEIL9 function as upstream regulators of carotenoid biosynthesis and the chlorophyll degradation pathway in banana ripening, respectively [19, 20]. However, the upstream transcriptional regulators of these TFs, and the connections among them, remain unclear. MADS and NAC are well recognized as two important classes of TFs contributing to fruit ripening [46, 47]. Genome ENCODE analyses reveal that NACs and MADSs form the dual-loop molecular circuit controlling banana ripening [48], but direct evidence of their interconnections is still lacking. Previous studies showed that MaMADS1, which is homologous to the tomato ripening regulator RIN, is necessary for banana fruit ripening [28], but its precise regulatory mechanisms, especially its target genes, remain elusive. The present study showed that MaMADS1 directly targeted the promoter of *MaNAC083* to inhibit its expression, which in turn attenuated MaNAC083's repression of the *MaACS1*, *MaACO1*, *MaACO4*, *MaACO5*, and *MaACO8* genes, and eventually promoted ethylene biosynthesis and fruit ripening (Figs 5 and6). These findings indicate that MaMADS1 and MaNAC083 constitute a transcriptional cascade to regulate ethylene biosynthesis genes. Moreover, ethylene induced the expression and promoter activity of *MaMADS1* (Supplementary Data Fig. S6A and B). These data also establish that ethylene inhibits MaNAC083-repressed ethylene biosynthesis by activating MaMADS1-mediated transcriptional repression of *MaNAC083*, which is a feedback regulation.

**Figure 7:**
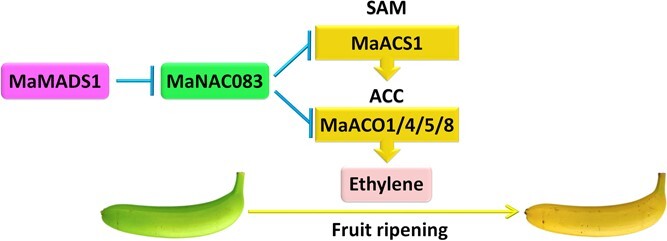
A proposed model for the transcriptional cascade function of MaMADS1–MaNAC083 in ethylene biosynthesis throughout banana ripening. MaMADS1 binds the *MaNAC083* promoter and represses its expression, which attenuates the MaNAC083-mediated repression of *MaACS1*, *MaACO1*, *MaACO4*, *MaACO5*, and *MaACO8*, and eventually promotes ethylene biosynthesis.

Based on the present findings, a working model is proposed to reveal the MaMADS1–MaNAC083 module that regulates ethylene biosynthesis during banana fruit ripening (Fig. 7). During postharvest ripening, *MaMADS1* gene expression is activated. MaMADS1 binds directly to the *MaNAC083* promoter and inhibits its expression. This negative regulation antagonizes MaNAC083's transcriptional repression of *MaACS1*, *MaACO1*, *MaACO4*, *MaACO5*, and *MaACO8*, and after this it induces ethylene biosynthesis. Our findings represent a transcriptional regulatory cascade, MaMADS1–MaNAC083–*MaACS1/MaACOs*, regulating ethylene biosynthesis during ripening, offering novel transcriptional insights into fruit ripening development. These findings may also contribute to different molecular approaches to improve the storability of fleshy fruits.

## Materials and methods

### Plant materials and samples

Pre-climacteric bananas (*M. acuminata*, AAA group, cv. ‘Cavendish’) at 75–80% maturity were gathered from a plantation near Guangzhou, China. The fruits were then subjected to ripening by incubation with 100 μL L^−1^ ethylene for 18 h. As Zhu *et al*. [25] described, the fruits were ripened at 20°C for 7 days in closed polyethylene bags (0.01 mm thickness). CI, firmness and ethylene generation were monitored at all sampling points, adopting a previously published method [49]. The samples were snap-frozen in liquid nitrogen and placed in cold storage at −80°C until further use.

### Gene expression analysis

Total banana fruit RNA was isolated utilizing the hot borate technique [44]. Using the Hieff^®^ qPCR SYBR Green Master Mix Kit (Yeasen), qRT–PCR reactions were carried out via CFX96 real-time PCR (Bio-Rad, USA). As Chen *et al*. [50] indicated, primer premier (version 5.0) was used to build primer sequences, and MaRPS4 was employed as an internal reference gene.

### Yeast one-hybrid assay

Y1H screening was done utilizing the Matchmaker™ Gold Yeast One-Hybrid System (Clontech, Takara, Japan). The short fragment of the *MaACS1* promoter (−499 bp upstream of ATG) or *MaNAC083* promoter (−676 bp upstream of ATG) was cloned into the pAbAi vector to generate the bait plasmid, followed by linearization and transformation into the Y1H Gold strain to produce the bait-specific reporter strain, which was preceded by screening a cDNA library of banana fruits. *MaNAC083* or *MaMADS1* coding sequences were cloned into the pGADT7 prey vector and introduced into bait-reporter yeast strains for re-transformation. According to the manufacturer's guidelines, DNA–protein interactions were assessed based on the co-transformants' ability to grow on SD/−Leu media with AbA.

### Electrophoretic mobility shift assay

The MaNAC083 coding sequence was inserted in-frame into pGEX-4 T-1 vector with a GST tag. Using glutathione–Sepharose 4B beads, GST-MaNAC083 fusion protein in *Escherichia coli BM Rosetta* (DE3) was purified. Fragments containing NACRS in the promoter of *MaACS1*, *MaACO1*, *MaACO4*, *MaACO5*, and *MaACO8* were manufactured (Sangon Biotech, Shanghai, China), and labeled with biotin at the 5′ end. As stated in our previously published work [30], EMSA was performed using a LightShift Chemiluminescent EMSA kit (Thermo Scientific). After incubating biotin-labeled probes with GST-MaNAC083 recombinant protein, the bound and free probes were determined on a native acrylamide gel. Unlabeled probes were used as competitors, whereas GST protein acted as a negative regulator.

### Promoter activity and subcellular localization assay

Using a previously described technique [30], promoter activity and subcellular localization assays were performed on tobacco BY-2 suspension culture cell protoplasts. Briefly, *MaNAC083* or *MaMADS1* promoter was extracted and placed in the pGreenII 0800-LUC reporter vector to induce the firefly luciferase reporter (LUC) gene. CaMV35S-REN/*MaNAC083pro*-LUC or CaMV35S-REN/*MaMADS1pro*-LUC constructs were transferred into tobacco protoplasts using a PEG-mediated method. Using a dual LUC assay kit (Promega), LUC and *Renilla* luciferase (REN) activities of transformed protoplasts supplemented with 0 (–ETH) or 10 μL L^−1^ ethylene (+ETH) were measured.

MaNAC083 and MaMADS1 coding sections were transferred into the pBI221-GFP vector to bind with the GFP reporter gene utilizing NLS-mCherry [30] as a nuclear marker for subcellular localization. Co-transfection of MaNAC083-GFP or MaMADS1-GFP and NLS-mCherry into BY-2 protoplasts was performed as previously described. A Zeiss Axioskop 2 Plus fluorescence microscope was utilized to record GFP and mCherry fluorescence signals.

### Dual-luciferase transient expression assay

Transcriptional capability was accessed by inserting full-length *MaNAC083* or *MaMADS1* coding sequence into the pGreenII 62SK-BD vector as an effector. The double-reporter vector featured an LUC gene controlled by five GAL4-binding element copies. The effector and recombinant reporter constructs were co-transfected into tobacco leaves, as in our prior research [34]. As reported in the promoter activity experiment, LUC and REN activities were assessed 60 h after injection.

To evaluate MaNAC083 or MaMADS1's influence on promoters of target genes, cloning of *MaNAC083 or MaMADS1* was performed into pGreenII 62-SK vector effector, and cloning of target gene promoters was performed into pGreenII 0800-LUC reporter vector [51]. Tobacco was co-transformed with an effector and a reporter plasmid utilizing *Agrobacterium tumefaciens* strain EHA105 and pSoup vector. The LUC/REN ratio reflected target promoter transcriptional repression. Each pair had six transient assay replicates.

### Transient overexpression and silencing analysis in banana fruit

As previously mentioned, transient overexpression investigation and silencing were done in banana fruit [26, 30]. The open reading frames of *MaNAC083* or *MaMADS1* were cloned into the pCXUN-HA vector, respectively. For vector construction in VIGS, *MaNAC083* or *MaMADS1* was cloned into the pTRV2 vector. Cells of the *A. tumefaciens* strain EHA105 carrying recombinant pTRV2 and pTRV1 were combined at a 1:1 ratio. The pulp was injected with *A. tumefaciens* harboring the constructed plasmids. On day 1 following inoculation, the transformed fruits were exposed to 100 μL L^−1^ ethylene and kept at 20°C for 5 days. The samples were collected to evaluate ethylene generation, gene expression, and protein accumulation.

### Statistical analysis

SPSS (v.19.0, SPSS Inc.) was used for execute statistical analysis. Data were represented as mean ± standard error of three or six independent biological replicates. Analysis of variance (ANOVA) or Student's *t*-test was utilized, as appropriate, to identify statistically significant differences between samples.

### Primers

Supplementary Data Table S2 lists the primers employed for this study.

## Acknowledgements

This study was supported by the National Key Research and Development Program of China (grant no. 2022YFD2100103), the National Natural Science Foundation of China (grant no. 32072279), and the China Agriculture Research System of MOF and MARA (grant no. CARS-31).

## Author contributions

W.S. conceived and designed the study, while W.W. performed experiments alongside W.S. Data analysis was carried out by a team consisting of W.W., Y.Y.Y, C.J.W, J.F.K., W.J.L., and W.S. The manuscript writing and revision were done by J.Y.C., W.S., and W.W.; all authors participated in manuscript discussions.

## Data availability

All study data are incorporated in the submitted article.

## Conflict of interest

None declared.

## Supplementary data

Supplementary data is available at Horticulture Research online.

## Supplementary Material

Web_Material_uhad177

## References

[1] Binder BM . Ethylene signaling in plants. *J Biol Chem*. 2020;295:7710–25 32332098 10.1074/jbc.REV120.010854 PMC7261785

[2] Kou X , Zhou J, Wu CE et al. The interplay between ABA/ethylene and NAC TFs in tomato fruit ripening: a review. *Plant Mol Biol*. 2021;106:223–38 33634368 10.1007/s11103-021-01128-w

[3] Boller T , Herner RC, Kende H. Assay for and enzymatic formation of an ethylene precursor, 1-aminocyclopropane-1-carboxylic acid. *Planta*. 1979;145:293–303 24317737 10.1007/BF00454455

[4] Adams DO , Yang SF. Ethylene biosynthesis: identification of 1-aminocyclopropane-1-carboxylic acid as an intermediate in the conversion of methionine to ethylene. *Proc Natl Acad Sci USA*. 1979;76:170–4 16592605 10.1073/pnas.76.1.170 PMC382898

[5] Pattyn J , Vaughan-Hirsch J, van de Poel B. The regulation of ethylene biosynthesis: a complex multilevel control circuitry. *New Phytol*. 2021;229:770–82 32790878 10.1111/nph.16873 PMC7820975

[6] Strader L , Weijers D, Wagner D. Plant transcription factors – being in the right place with the right company. *Curr Opin Plant Biol*. 2022;65:102136 34856504 10.1016/j.pbi.2021.102136 PMC8844091

[7] Giovannoni JJ . Fruit ripening mutants yield insights into ripening control. *Curr Opin Plant Biol*. 2007;10:283–9 17442612 10.1016/j.pbi.2007.04.008

[8] Seymour GB , Granell A. Fruit development and ripening preface. *J Exp Bot*. 2014;65:4489–90 25221812 10.1093/jxb/eru307 PMC4115256

[9] Ito Y , Kitagawa M, Ihashi N et al. DNA-binding specificity, transcriptional activation potential, and the rin mutation effect for the tomato fruit-ripening regulator RIN. *Plant J*. 2008;55:212–23 18363783 10.1111/j.1365-313X.2008.03491.x

[10] Li L , Wang X, Zhang X et al. Unraveling the target genes of RIN transcription factor during tomato fruit ripening and softening. *J Sci Food Agric*. 2017;97:991–1000 27247090 10.1002/jsfa.7825

[11] Gao Y , Wei W, Fan ZQ et al. Re-evaluation of the nor mutation and the role of the NAC-NOR transcription factor in tomato fruit ripening. *J Exp Bot*. 2020;71:3560–74 32338291 10.1093/jxb/eraa131 PMC7307841

[12] Huang S , Sawaki T, Takahashi A et al. Melon EIN3-like transcription factors (CmEIL1 and CmEIL2) are positive regulators of an ethylene- and ripening-induced 1-aminocyclopropane-1-carboxylic acid oxidase gene (*CM-ACO1*). *Plant Sci*. 2010;178:251–7

[13] Han YC , Kuang JF, Chen JY et al. Banana transcription factor MaERF11 recruits histone deacetylase MaHDA1 and represses the expression of MaACO1 and expansins during fruit ripening. *Plant Physiol*. 2016;171:1070–84 27208241 10.1104/pp.16.00301 PMC4902611

[14] Espley RV , Leif D, Plunkett B et al. Red to brown: an elevated anthocyanic response in apple drives ethylene to advance maturity and fruit flesh browning. *Front*. *Plant Sci*. 2019;10:1248 10.3389/fpls.2019.01248 PMC6794385 31649709

[15] Sadka A , Qin Q, Feng J et al. Ethylene response of plum ACC synthase 1 (ACS1) promoter is mediated through the binding site of abscisic acid insensitive 5 (ABI5). *Plants (Basel)*. 2019;8:117 31052513 10.3390/plants8050117 PMC6572237

[16] Aurore G , Parfait B, Fahrasmane L. Bananas, raw materials for making processed food products. Trends Food Sci Technol. 2009;20:78–91

[17] Lescot T . Banana genetic diversity: estimated world production by type of banana. FruiTrop. 2020;269:98–102

[18] Xiao YY , Kuang JF, Qi XN et al. A comprehensive investigation of starch degradation process and identification of a transcriptional activator MabHLH6 during banana fruit ripening. Plant Biotechnol J. 2018;16:151–64 28500777 10.1111/pbi.12756 PMC5785343

[19] Inaba A , Liu X, Yokotani N et al. Differential feedback regulation of ethylene biosynthesis in pulp and peel tissues of banana fruit. J Exp Bot. 2007;58:1047–57 17185740 10.1093/jxb/erl265

[20] Xiao YY , Chen JY, Kuang JF et al. Banana ethylene response factors are involved in fruit ripening through their interactions with ethylene biosynthesis genes. J Exp Bot. 2013;64:2499–510 23599278 10.1093/jxb/ert108 PMC3654433

[21] Wang Z , Miao HX, Liu JH et al. Musa balbisiana genome reveals subgenome evolution and functional divergence. Nat Plants. 2019;5:810–21 31308504 10.1038/s41477-019-0452-6 PMC6784884

[22] Liu X , Shiomi S, Nakatsuka A et al. Characterization of ethylene biosynthesis associated with ripening in banana fruit. Plant Physiol. 1999;121:1257–65 10594112 10.1104/pp.121.4.1257 PMC59492

[23] Jourda C , Cardi C, Mbéguié-A-Mbéguié D et al. Expansion of banana (*Musa acuminata*) gene families involved in ethylene biosynthesis and signalling after lineage-specific whole-genome duplications. New Phytol. 2014;202:986–1000 24716518 10.1111/nph.12710

[24] Wei W , YangYY, ChenJYet al. MaNAC029 modulates ethylene biosynthesis and fruit quality and undergoes MaXB3-mediated proteasomal degradation during banana ripening. J Adv Res. 202210.1016/j.jare.2022.12.00436529351

[25] Zhu LS , Chen L, Wu CJ et al. Methionine oxidation and reduction of the ethylene signaling component MaEIL9 are involved in banana fruit ripening. J Integr Plant Biol. 2023;65:150–66 36103229 10.1111/jipb.13363

[26] Wei W , Yang YY, Lakshmanan P et al. Proteasomal degradation of MaMYB60 mediated by the E3 ligase MaBAH1 causes high temperature-induced repression of chlorophyll catabolism and green ripening in banana. Plant Cell. 2023;35:1408–28 36748200 10.1093/plcell/koad030 PMC10118274

[27] Cenci A , Guignon V, Roux N et al. Genomic analysis of NAC transcription factors in banana (*Musa acuminata*) and definition of NAC orthologous groups for monocots and dicots. Plant Mol Biol. 2014;85:63–80 24570169 10.1007/s11103-013-0169-2 PMC4151281

[28] Elitzur T , Yakir E, Quansah L et al. Banana MaMADS transcription factors are necessary for fruit ripening and molecular tools to promote shelf-life and food security. Plant Physiol. 2016;171:380–91 26956665 10.1104/pp.15.01866 PMC4854685

[29] Tilly JJ , Allen DW, Jack T. The CArG boxes in the promoter of the Arabidopsis floral organ identity gene APETALA3 mediate diverse regulatory effects. Development. 1998;125:1647–57 9521903 10.1242/dev.125.9.1647

[30] Shan W , Kuang JF, Wei W et al. MaXB3 modulates MaNAC2, MaACS1, and MaACO1 stability to repress ethylene biosynthesis during banana fruit ripening. Plant Physiol. 2020;184:1153–71 32694134 10.1104/pp.20.00313 PMC7536691

[31] Ebrahimi A , Zabihzadeh Khajavi M, Ahmadi S et al. Novel strategies to control ethylene in fruit and vegetables for extending their shelf life: a review. Int J Environ Sci Technol. 2022;19:4599–610

[32] Yang YY , Shan W, Yang TW et al. MaMYB4 is a negative regulator and a substrate of RING-type E3 ligases MaBRG2/3 in controlling banana fruit ripening. Plant J. 2022;110:1651–69 35395128 10.1111/tpj.15762

[33] Li XM , Wang XM, Zhang Y et al. Regulation of fleshy fruit ripening: from transcription factors to epigenetic modifications. Hortic Res. 2022;9:9 10.1093/hr/uhac013 PMC9035223 35147185

[34] Fan ZQ , Ba LJ, Shan W et al. A banana R2R3-MYB transcription factor MaMYB3 is involved in fruit ripening through modulation of starch degradation by repressing starch degradation-related genes and MabHLH6. Plant J. 2018;96:1191–205 30242914 10.1111/tpj.14099

[35] Kuang JF , Wu CJ, Guo YF et al. Deciphering transcriptional regulators of banana fruit ripening by regulatory network analysis. Plant Biotechnol J. 2021;19:477–89 32920977 10.1111/pbi.13477 PMC7955892

[36] Kim HJ , Nam HG, Lim PO. Regulatory network of NAC transcription factors in leaf senescence. Curr Opin Plant Biol. 2016;33:48–56 27314623 10.1016/j.pbi.2016.06.002

[37] Song LL , Wang JP, Yu YT et al. 1-Methylcyclopropene retards pak choi (Brassica rapa subsp. chinensis) yellowing via BcNAC055-, BcMYB44-, and BcOBF1-mediated regulation of the key chlorophyll degrading gene BcNYC1 during storage at 20 °C. Food Qual Saf. 2022;7:fyac075

[38] Jiang GX , Zeng J, Li ZW et al. Redox regulation of the NOR transcription factor is involved in the regulation of fruit ripening in tomato. Plant Physiol. 2020;183:671–85 32234754 10.1104/pp.20.00070 PMC7271799

[39] Gao Y , Zhu N, Zhu XF et al. Diversity and redundancy of the ripening regulatory networks revealed by the fruit ENCODE and the new CRISPR/Cas9 CNR and NOR mutants. Hortic Res. 2019;6:39 30774962 10.1038/s41438-019-0122-x PMC6370854

[40] Kumar R , Tamboli V, Sharma R et al. NAC-NOR mutations in tomato Penjar accessions attenuate multiple metabolic processes and prolong the fruit shelf life. Food Chem. 2018;259:234–44 29680049 10.1016/j.foodchem.2018.03.135

[41] Ma NN , Feng HL, Meng X et al. Overexpression of tomato SlNAC1 transcription factor alters fruit pigmentation and softening. BMC Plant Biol. 2014;14:351 25491370 10.1186/s12870-014-0351-y PMC4272553

[42] Gao Y , Wei W, Zhao X et al. A NAC transcription factor, NOR-like1, is a new positive regulator of tomato fruit ripening. Hortic Res. 2018;5:75 30588320 10.1038/s41438-018-0111-5 PMC6303401

[43] Wu YY , Liu XF, Fu BL et al. Methyl jasmonate enhances ethylene synthesis in kiwifruit by inducing NAC genes that activate ACS1. J Agric Food Chem. 2020;68:3267–76 32101430 10.1021/acs.jafc.9b07379

[44] Yang SD , Seo PJ, Yoon HK et al. The Arabidopsis NAC transcription factor VNI2 integrates abscisic acid signals into leaf senescence via the COR/RD genes. Plant Cell. 2011;23:2155–68 21673078 10.1105/tpc.111.084913 PMC3160032

[45] Wu CJ , Shan W, Liu XC et al. Phosphorylation of transcription factor bZIP21 by MAP kinase MPK6-3 enhances banana fruit ripening. Plant Physiol. 2022;188:1665–85 34792564 10.1093/plphys/kiab539 PMC8896643

[46] Li S , Chen K, Grierson D. A critical evaluation of the role of ethylene and MADS transcription factors in the network controlling fleshy fruit ripening. New Phytol. 2019;221:1724–41 30328615 10.1111/nph.15545

[47] Liu GS , Li HL, Grierson D et al. NAC transcription factor family regulation of fruit ripening and quality: a review. Cell. 2022;11:525 10.3390/cells11030525 PMC8834055 35159333

[48] Lv P , Yu S, Zhu N et al. Genome encode analyses reveal the basis of convergent evolution of fleshy fruit ripening. Nat Plants. 2018;4:784–91 30250279 10.1038/s41477-018-0249-z

[49] Wei W , Chen JY, Zeng ZX et al. The ubiquitin e3 ligase MaLUL2 is involved in high temperature-induced green ripening in banana fruit. Int J Mol Sci. 2020;21:9386 33317166 10.3390/ijms21249386 PMC7763436

[50] Chen L , Zhong XY, Kuang JF et al. Validation of reference genes for RT-qPCR studies of gene expression in banana fruit under different experimental conditions. Planta. 2011;234:377–90 21505864 10.1007/s00425-011-1410-3

[51] Hellens RP , Allan AC, Friel EN et al. Transient expression vectors for functional genomics, quantification of promoter activity and RNA silencing in plants. Plant Methods. 2005;1:13 16359558 10.1186/1746-4811-1-13 PMC1334188

