# Supplementary material for: MaMADS1–MaNAC083 transcriptional regulatory cascade regulates ethylene biosynthesis during banana fruit ripening
Source: Hortic Res. 2023 Sep 8;10(10):uhad177. doi: 10.1093/hr/uhad177 (PMC10585711; doi:10.1093/hr/uhad177)
Supplement: Web_Material_uhad177 [file web_material_uhad177.zip › MaMADS1-MANAC083-Supplemental_Tables.pdf]

Table S1

List of ethylene biosynthesis genes between unripe and ripe banana fruit.

Gene ID	Gene name	Unripe-1_fpkm	Unripe-2_fpkm	Unripe-3_fpkm	Ripe-1_fpkm	Ripe-2_fpkm	Ripe-3_fpkm	Unripe_mean	Ripe_mean	log2(Fold change)	PValue	FDR	Description	KEGG_A_class	KEGG_B_class	Pathway	K_ID	GO Function	GO Process
Ma01_g06520	MaSAMS1	554.05	241.92	441.09	543.74	578.48	481.88	412.3533333	534.7	0.374848607	0.065507739	0.096630639	S-adenosylmethionine synthase 1	Metabolism;Metabolism;Metabolism;Metabolism	Global and overview maps;Global and overview maps;Global and overview maps;Amino acid metabolism	ko01100//Metabolic pathways;ko01110//Biosynthesis of secondary metabolites;ko01230//Biosynthesis of amino acids;ko00270//Cysteine and methionine metabolism	K00789;K00789;K00789;K00789	GO:0016765//transferase activity, transferring alkyl or aryl (other than methyl) groups;GO:0032550//purine ribonucleoside binding;GO:0043169//cation binding	GO:0009108//coenzyme biosynthetic process
Ma01_g09270	MaSAMS2	40.06	27.27	34.14	36.62	36.6	34.11	33.82333333	35.77666667	0.081000131	0.171730359	0.226130849	S-adenosylmethionine synthase 5-like	Metabolism;Metabolism;Metabolism;Metabolism	Global and overview maps;Global and overview maps;Global and overview maps;Amino acid metabolism	ko01100//Metabolic pathways;ko01110//Biosynthesis of secondary metabolites;ko01230//Biosynthesis of amino acids;ko00270//Cysteine and methionine metabolism	K00789;K00789;K00789;K00789	-	-
Ma02_g09900	MaSAMS4	1573.7	978.34	1199.69	77.48	78.78	63.85	1250.576667	73.37	-4.091259409	6.53E-41	1.49E-39	S-adenosylmethionine synthase 5	Metabolism;Metabolism;Metabolism;Metabolism	Global and overview maps;Global and overview maps;Global and overview maps;Amino acid metabolism	ko01100//Metabolic pathways;ko01110//Biosynthesis of secondary metabolites;ko01230//Biosynthesis of amino acids;ko00270//Cysteine and methionine metabolism	K00789;K00789;K00789;K00789	-	-
Ma03_g08390	MaSAMS5	6.78	3.5	6.55	4.41	5.74	6.09	5.61	5.413333333	-0.051483544	0.59251228	0.655608074	S-adenosylmethionine synthase-like	Metabolism;Metabolism;Metabolism;Metabolism	Global and overview maps;Global and overview maps;Global and overview maps;Amino acid metabolism	ko01100//Metabolic pathways;ko01110//Biosynthesis of secondary metabolites;ko01230//Biosynthesis of amino acids;ko00270//Cysteine and methionine metabolism	K00789;K00789;K00789;K00789	GO:0016765//transferase activity, transferring alkyl or aryl (other than methyl) groups;GO:0032550//purine ribonucleoside binding;GO:0043169//cation binding	GO:0009108//coenzyme biosynthetic process
Ma03_g12530	MaSAMS6	21.47	13.13	19.69	7.4	6.48	5.76	18.09666667	6.546666667	-1.466891554	0.000203699	0.000462607	S-adenosylmethionine synthase	Metabolism;Metabolism;Metabolism;Metabolism	Global and overview maps;Global and overview maps;Global and overview maps;Amino acid metabolism	ko01100//Metabolic pathways;ko01110//Biosynthesis of secondary metabolites;ko01230//Biosynthesis of amino acids;ko00270//Cysteine and methionine metabolism	K00789;K00789;K00789;K00789	-	-
Ma05_g23390	MaSAMS7	272.45	138.81	224.38	189.7	228.53	182.47	211.88	200.2333333	-0.081565251	0.566254217	0.631355134	S-adenosylmethionine synthase 1-like	Metabolism;Metabolism;Metabolism;Metabolism	Global and overview maps;Global and overview maps;Global and overview maps;Amino acid metabolism	ko01100//Metabolic pathways;ko01110//Biosynthesis of secondary metabolites;ko01230//Biosynthesis of amino acids;ko00270//Cysteine and methionine metabolism	K00789;K00789;K00789;K00789	GO:0016765//transferase activity, transferring alkyl or aryl (other than methyl) groups;GO:0032550//purine ribonucleoside binding;GO:0043169//cation binding	GO:0009108//coenzyme biosynthetic process
Ma05_g28480	MaSAMS9	0	0.08	0.1	0	0.1	0.33	0.06	0.143333333	1.256339753	0.373728181	0.443239607	S-adenosylmethionine synthase 3	Metabolism;Metabolism;Metabolism;Metabolism	Global and overview maps;Global and overview maps;Global and overview maps;Amino acid metabolism	ko01100//Metabolic pathways;ko01110//Biosynthesis of secondary metabolites;ko01230//Biosynthesis of amino acids;ko00270//Cysteine and methionine metabolism	K00789;K00789;K00789;K00789	-	-
Ma07_g02100	MaSAMS10	0	0	0	0.26	0.05	0	0.001	0.103333333	6.691161905	0.160888268	0.213841385	S-adenosylmethionine synthase 5-like	Metabolism;Metabolism;Metabolism;Metabolism	Global and overview maps;Global and overview maps;Global and overview maps;Amino acid metabolism	ko01100//Metabolic pathways;ko01110//Biosynthesis of secondary metabolites;ko01230//Biosynthesis of amino acids;ko00270//Cysteine and methionine metabolism	K00789;K00789;K00789;K00789	GO:0016765//transferase activity, transferring alkyl or aryl (other than methyl) groups;GO:0032550//purine ribonucleoside binding;GO:0043169//cation binding	GO:0009108//coenzyme biosynthetic process
Ma08_g06060	MaSAMS11	2047.23	1052.5	1717.07	101.82	109.12	90.57	1605.6	100.5033333	-3.997797266	4.51E-32	6.98E-31	S-adenosylmethionine synthase	Metabolism;Metabolism;Metabolism;Metabolism	Global and overview maps;Global and overview maps;Global and overview maps;Amino acid metabolism	ko01100//Metabolic pathways;ko01110//Biosynthesis of secondary metabolites;ko01230//Biosynthesis of amino acids;ko00270//Cysteine and methionine metabolism	K00789;K00789;K00789;K00789	-	-
Ma11_g24630	MaSAMS12	0.07	0.31	0	0	0	0	0.126666667	0.001	-6.984893108	0.279293767	1	S-adenosylmethionine synthase 3-like	Metabolism;Metabolism;Metabolism;Metabolism	Global and overview maps;Global and overview maps;Global and overview maps;Amino acid metabolism	ko01100//Metabolic pathways;ko01110//Biosynthesis of secondary metabolites;ko01230//Biosynthesis of amino acids;ko00270//Cysteine and methionine metabolism	K00789;K00789;K00789;K00789	GO:0016765//transferase activity, transferring alkyl or aryl (other than methyl) groups;GO:0032550//purine ribonucleoside binding;GO:0043169//cation binding	GO:0009108//coenzyme biosynthetic process
Ma04_g35640	MaACS1	0.08	0.11	0.17	126.96	160.18	168.08	0.12	151.74	10.30435132	1.63E-92	1.72E-90	1-aminocyclopropane-1-carboxylate synthase	Metabolism;Metabolism;Metabolism	Global and overview maps;Global and overview maps;Amino acid metabolism	ko01100//Metabolic pathways;ko01110//Biosynthesis of secondary metabolites;ko00270//Cysteine and methionine metabolism	K01762;K01762;K01762	-	-
Ma02_g10500	MaACS2	0	0	0	0	0	0	0.001	0.001	0	1	1	1-aminocyclopropane-1-carboxylate synthase 3	Metabolism;Metabolism;Metabolism	Global and overview maps;Global and overview maps;Amino acid metabolism	ko01100//Metabolic pathways;ko01110//Biosynthesis of secondary metabolites;ko00270//Cysteine and methionine metabolism	K01762;K01762;K01762	-	-
Ma01_g07800	MaACS3	0.28	0	0.05	0.12	0.05	0	0.11	0.056666667	-0.956931278	0.690882764	0.745133231	1-aminocyclopropane-1-carboxylate synthase 1	Metabolism;Metabolism;Metabolism	Global and overview maps;Global and overview maps;Amino acid metabolism	ko01100//Metabolic pathways;ko01110//Biosynthesis of secondary metabolites;ko00270//Cysteine and methionine metabolism	K01762;K01762;K01762	GO:0043168//anion binding	GO:0008152//metabolic process
Ma03_g27050	MaACS4	0	0	0	0	0.09	0	0.001	0.03	4.906890596	0.635101243	1	1-aminocyclopropane-1-carboxylate synthase 3	Metabolism;Metabolism;Metabolism	Global and overview maps;Global and overview maps;Amino acid metabolism	ko01100//Metabolic pathways;ko01110//Biosynthesis of secondary metabolites;ko00270//Cysteine and methionine metabolism	K01762;K01762;K01762	-	-
Ma05_g08580	MaACS5	0	0	0	0	0	0	0.001	0.001	0	1	1	1-aminocyclopropane-1-carboxylate synthase 3-like	Metabolism;Metabolism;Metabolism	Global and overview maps;Global and overview maps;Amino acid metabolism	ko01100//Metabolic pathways;ko01110//Biosynthesis of secondary metabolites;ko00270//Cysteine and methionine metabolism	K01762;K01762;K01762	GO:0043168//anion binding	GO:0008152//metabolic process
Ma04_g24230	MaACS6	0	0	0	0	0	0	0.001	0.001	0	1	1	1-aminocyclopropane-1-carboxylate synthase 3-like	Metabolism;Metabolism;Metabolism	Global and overview maps;Global and overview maps;Amino acid metabolism	ko01100//Metabolic pathways;ko01110//Biosynthesis of secondary metabolites;ko00270//Cysteine and methionine metabolism	K01762;K01762;K01762	GO:0043168//anion binding	GO:0008152//metabolic process
Ma04_g01260	MaACS7	0	0	0	0	0	0	0.001	0.001	0	1	1	1-aminocyclopropane-1-carboxylate synthase 8-like	Metabolism;Metabolism;Metabolism	Global and overview maps;Global and overview maps;Amino acid metabolism	ko01100//Metabolic pathways;ko01110//Biosynthesis of secondary metabolites;ko00270//Cysteine and methionine metabolism	K01762;K01762;K01762	-	-
Ma09_g19150	MaACS8	0	0	0	0	0	0	0.001	0.001	0	1	1	1-aminocyclopropane-1-carboxylate synthase	Metabolism;Metabolism;Metabolism	Global and overview maps;Global and overview maps;Amino acid metabolism	ko01100//Metabolic pathways;ko01110//Biosynthesis of secondary metabolites;ko00270//Cysteine and methionine metabolism	K01762;K01762;K01762	-	-
Ma04_g31490	MaACS9	0	0	0	0	0	0	0.001	0.001	0	1	1	1-aminocyclopropane-1-carboxylate synthase	Metabolism;Metabolism;Metabolism	Global and overview maps;Global and overview maps;Amino acid metabolism	ko01100//Metabolic pathways;ko01110//Biosynthesis of secondary metabolites;ko00270//Cysteine and methionine metabolism	K01762;K01762;K01762	-	-
Ma04_g37400	MaACS10	0	0	0	0	0	0	0.001	0.001	0	1	1	ACC syntase [Musa acuminata AAA Group]	Metabolism;Metabolism;Metabolism	Global and overview maps;Global and overview maps;Amino acid metabolism	ko01100//Metabolic pathways;ko01110//Biosynthesis of secondary metabolites;ko00270//Cysteine and methionine metabolism	K01762;K01762;K01762	GO:0043168//anion binding	GO:0008152//metabolic process
Ma10_g27510	MaACS11	0	0	0	0	0	0	0.001	0.001	0	1	1	1-aminocyclopropane-1-carboxylate synthase 7-like isoform X1	Metabolism;Metabolism;Metabolism	Global and overview maps;Global and overview maps;Amino acid metabolism	ko01100//Metabolic pathways;ko01110//Biosynthesis of secondary metabolites;ko00270//Cysteine and methionine metabolism	K01762;K01762;K01762	GO:0043168//anion binding	GO:0008152//metabolic process
Ma05_g13700	MaACS12	165.07	215.9	169	2.58	2.33	3.1	183.3233333	2.67	-6.101406871	1.60E-214	2.04E-211	probable aminotransferase ACS12	-	-	-	-	GO:0008483//transaminase activity;GO:0043168//anion binding	GO:0019752//carboxylic acid metabolic process
Ma03_g12030	MaACS13	1.05	0.65	0.55	0.39	0.66	0.29	0.75	0.446666667	-0.747692001	0.475039292	0.543285454	probable aminotransferase ACS12	-	-	-	-	GO:0043168//anion binding	GO:0008152//metabolic process
Ma01_g12130	MaACS14	3.97	6.22	3.54	8.4	9.68	9.64	4.576666667	9.24	1.013595632	1.75E-10	7.49E-10	probable aminotransferase ACS12	-	-	-	-	GO:0008483//transaminase activity;GO:0043168//anion binding	GO:0019752//carboxylic acid metabolic process
Ma07_g19730	MaACO1	932.96	679.98	906.45	6387.9	6080.12	6021.71	839.7966667	6163.243333	2.875577785	9.26E-36	1.71E-34	1-aminocyclopropane-1-carboxylate oxidase	Metabolism;Metabolism;Metabolism	Global and overview maps;Global and overview maps;Amino acid metabolism	ko01100//Metabolic pathways;ko01110//Biosynthesis of secondary metabolites;ko00270//Cysteine and methionine metabolism	K05933;K05933;K05933	-	-
Ma00_g04770	MaACO2	0	0	0	0	0	0	0.001	0.001	0	1	1	1-aminocyclopropane-1-carboxylate oxidase 3-like	Metabolism;Metabolism;Metabolism	Global and overview maps;Global and overview maps;Amino acid metabolism	ko01100//Metabolic pathways;ko01110//Biosynthesis of secondary metabolites;ko00270//Cysteine and methionine metabolism	K05933;K05933;K05933	GO:0016705//oxidoreductase activity, acting on paired donors, with incorporation or reduction of molecular oxygen;GO:0043169//cation binding	GO:0044710//single-organism metabolic process
Ma06_g14410	MaACO3	0.53	0.63	0.47	0.62	0.28	0.19	0.543333333	0.363333333	-0.580543829	0.753955064	0.801098849	1-aminocyclopropane-1-carboxylate oxidase-like	Metabolism;Metabolism;Metabolism	Global and overview maps;Global and overview maps;Amino acid metabolism	ko01100//Metabolic pathways;ko01110//Biosynthesis of secondary metabolites;ko00270//Cysteine and methionine metabolism	K05933;K05933;K05933	-	-
Ma01_g11540	MaACO4	0.07	0.21	0.06	95.71	86.47	64.75	0.113333333	82.31	9.504351661	7.03E-39	1.49E-37	1-aminocyclopropane-1-carboxylate oxidase 1-like isoform X2	Metabolism;Metabolism;Metabolism	Global and overview maps;Global and overview maps;Amino acid metabolism	ko01100//Metabolic pathways;ko01110//Biosynthesis of secondary metabolites;ko00270//Cysteine and methionine metabolism	K05933;K05933;K05933	GO:0016705//oxidoreductase activity, acting on paired donors, with incorporation or reduction of molecular oxygen;GO:0043169//cation binding	GO:0044710//single-organism metabolic process
Ma05_g09360	MaACO5	0	0	0	1.4	1.91	2.08	0.001	1.796666667	10.81110706	1.91E-08	6.72E-08	1-aminocyclopropane-1-carboxylate oxidase 1	Metabolism;Metabolism;Metabolism	Global and overview maps;Global and overview maps;Amino acid metabolism	ko01100//Metabolic pathways;ko01110//Biosynthesis of secondary metabolites;ko00270//Cysteine and methionine metabolism	K05933;K05933;K05933	GO:0016491//oxidoreductase activity	-
Ma06_g02600	MaACO6	0.08	0.11	0.07	0.94	0.61	0.85	0.086666667	0.8	3.206450877	0.0008665	0.001802981	1-aminocyclopropane-1-carboxylate oxidase 1-like	Metabolism;Metabolism;Metabolism	Global and overview maps;Global and overview maps;Amino acid metabolism	ko01100//Metabolic pathways;ko01110//Biosynthesis of secondary metabolites;ko00270//Cysteine and methionine metabolism	K05933;K05933;K05933	-	-
Ma06_g14370	MaACO7	0.07	0.11	0.06	0.1	0.06	0	0.08	0.053333333	-0.584962501	0.95963333	0.969362682	1-aminocyclopropane-1-carboxylate oxidase-like	Metabolism;Metabolism;Metabolism	Global and overview maps;Global and overview maps;Amino acid metabolism	ko01100//Metabolic pathways;ko01110//Biosynthesis of secondary metabolites;ko00270//Cysteine and methionine metabolism	K05933;K05933;K05933	-	-
Ma10_g16100	MaACO8	0	0.33	0.04	2.26	1.73	1.46	0.123333333	1.816666667	3.880659054	1.48E-09	5.78E-09	1-aminocyclopropane-1-carboxylate oxidase-like	Metabolism;Metabolism;Metabolism	Global and overview maps;Global and overview maps;Amino acid metabolism	ko01100//Metabolic pathways;ko01110//Biosynthesis of secondary metabolites;ko00270//Cysteine and methionine metabolism	K05933;K05933;K05933	-	-
Ma06_g14420	MaACO9	0	0.27	0.19	0	0	0.27	0.153333333	0.09	-0.768674454	0.844165028	0.876751902	1-aminocyclopropane-1-carboxylate oxidase-like	Metabolism;Metabolism;Metabolism	Global and overview maps;Global and overview maps;Amino acid metabolism	ko01100//Metabolic pathways;ko01110//Biosynthesis of secondary metabolites;ko00270//Cysteine and methionine metabolism	K05933;K05933;K05933	-	-
Ma06_g14430	MaACO10	0	0	0	0.05	0	0	0.001	0.016666667	4.058893689	0.830102192	1	1-aminocyclopropane-1-carboxylate oxidase-like	Metabolism;Metabolism;Metabolism	Global and overview maps;Global and overview maps;Amino acid metabolism	ko01100//Metabolic pathways;ko01110//Biosynthesis of secondary metabolites;ko00270//Cysteine and methionine metabolism	K05933;K05933;K05933	-	-
Ma06_g14400	MaACO11	0.49	0.24	0	0.88	0.4	0	0.243333333	0.426666667	0.810175441	0.42085281	0.490440525	1-aminocyclopropane-1-carboxylate oxidase-like, partial	Metabolism;Metabolism;Metabolism	Global and overview maps;Global and overview maps;Amino acid metabolism	ko01100//Metabolic pathways;ko01110//Biosynthesis of secondary metabolites;ko00270//Cysteine and methionine metabolism	K05933;K05933;K05933	-	-
Ma06_g14390	MaACO12	0.15	0.45	0	0.11	0.13	0.39	0.2	0.21	0.070389328	0.743468896	0.792002575	1-aminocyclopropane-1-carboxylate oxidase-like, partial	Metabolism;Metabolism;Metabolism	Global and overview maps;Global and overview maps;Amino acid metabolism	ko01100//Metabolic pathways;ko01110//Biosynthesis of secondary metabolites;ko00270//Cysteine and methionine metabolism	K05933;K05933;K05933	-	-

Table S2
Summary of primers used in this study.

Assay	Primer sequences	Restriction Site
qRT-PCR	*MaRPS4-F: TGAGAGTGGCTTGACCCTGA*	
	MaRPS4-R: GTGACATTTAGTCGTCTGCTGG	
	MaACS1-F: ACAAGTTCAAGATCACCCAAGC	
	MaACS1-R: AGTGCATCCTTTTCTCGTTGAC	
	MaACO1-F: CCGATCTTGATGACCAGTAT	
	MaACO1-R: AGAGAAGGCTTTCTTCAGGT	
	MaACO4-F: ATGTCGATCGCCTCCTTCTA	
	MaACO4-R: GGCCTTCATTGCTTCAAACC	
	MaACO5-F: GGAGTTCGAGAGTGAGGAGA	
	MaACO5-R: CCACAGCTACTCCATGGTTC	
	MaACO8-F: CTTTGGCCCAGATCGCTAAT	
	MaACO8-R: CAGTCTGTAGCACTCGGAAC	
	MaSAMS4-F: GATGAGACACCTGAGCTGAT	
	MaSAMS4-R: ATTCTGGTACTCGACCGTAA	
	MaSAMS11-F: TTGTTGACACTTACGGAACA	
	MaSAMS11-R: AAATGTCCATAAGCTGCTGT	
	MaACS12-F: GAAATGTGACGGATACCCTA	
	MaACS12-R: ATGGATCAAACGAGACTGAC	
	MaNAC083-F: ACTGGGTGCTCTGTCGCATCTTTAAG	
	MaNAC083-R: TGTGCCGGAACTGGTTTCCTCTC	
	MaMADS1-F: GTGAAGCGAACAAGGGTCTCAGAAG	
	MaMADS1-R: GGGTTGACACTCCAATGGGTAGAATAAC	
Transient expression	*MaMADS1-62SK-F:AggatccATGGGGAGGGGGAGGGTG*	*Bam* H I
	MaMADS1-62SK-R: AgaattcTTCCAGCCATGCAGGCATAT	*Eco* R I
	MaMADS1-62SK-BD-F: AggatccATGGGGAGGGGGAGGGTG	*Bam* H I
	MaMADS1-62SK-BD-R:AgaattcTTCCAGCCATGCAGGCATAT	*Eco* R I
	MaMADS1pro-LUCReporter-F: AggtaccTTTGACTCAAGTAAACACTCTTCTT	*Kpn* I
	MaMADS1pro- LUCReporter-R: AccatggCGCGATCCCGCCCCGCCCCGGGCCA	*Nco* I
	MaNAC083-62SK-F: AggatccATGGATACGAAGCCGAGTGTTGTCGC	*Bam* H I
	MaNAC083-62SK-R: AgaattcCTACGGAAGTGATGTGCCGGAACTGG	*Eco* R I
	MaNAC083-62SK-BD-F: AggatccATGGATACGAAGCCGAGTGTTGTCGC	*Bam* H I
	MaNAC083-62SK-BD-R:AgaattcCTACGGAAGTGATGTGCCGGAACTGG	*Eco* R I
	MaNAC083pro-LUCReporter-F: AggtaccAAATTATTTGCTTTTAAGTATATTT	*Kpn* I
	MaNAC083pro- LUCReporter-R: AccatggATGAAACACGGACCAGGAAGCAGAG	*Nco* I
	MaACS1pro-LUCReporter-F: AggtaccAATCATTCACATGATCTTAACGTTA	*Kpn* I
	MaACS1pro- LUCReporter-R: AccatggGTGACCCGTTATCTCAGGTACG	*Nco* I
	MaACO1pro-LUCReporter-F: AggtaccTGGAGGTGTGTTTTCCGG	*Kpn* I
	MaACO1pro- LUCReporter-R: AccatggGACACGCTCTTTCTTCCCTGA	*Nco* I
	MaACO4pro-LUCReporter-F: AggtaccAGGAGAATCACAACGCACCT	*Kpn* I
	MaACO4pro- LUCReporter-R: AccatggTTCCTTTGTCTATTCCCACTCG	*Nco* I
	MaACO5pro-LUCReporter-F: AggtaccACTTGATCTTTAAATGGTCACTGTT	*Kpn* I
	MaACO5pro- LUCReporter-R: AccatggGCTTTGCTCCTCTCCTTGA	*Nco* I
	MaACO8pro-LUCReporter-F: AggtaccCTTCCCCCATGTGGCC	*Kpn* I
	MaACO8pro- LUCReporter-R: AccatggCCTGCCTCGATCTACAGAAG	*Nco* I
	MaNAC083-pCXUN-HA-F: AtgttccagattacgctATGGATACGAAGCCGAGTGTTGTCGC	*Xcm* I
	MaNAC083-pCXUN-HA-R: AaatgtttgaacgatcCTACGGAAGTGATGTGCCGGAACTGG	*Xcm* I
	MaMADS1-pCXUN-HA-F: AtgttccagattacgctATGGGGAGGGGGAGGGTG	*Xcm* I
	MaMADS1-pCXUN-HA-R: AaatgtttgaacgatcTTCCAGCCATGCAGGCATAT	*Xcm* I
	MaNAC083-pTRV2-F: AggatccATGGATACGAAGCCGAGTGTTGTCGC	*Bam* H I
	MaNAC083-pTRV2-R: AcccgggGTCCTTCCCCGTGGCC	*Sma* I
	MaMADS1-pTRV2-F:AggatccATGGGGAGGGGGAGGGTG	*Bam* H I
	MaMADS1-pTRV2-R: AcccgggAAGTTTTAAGTACTCTTGACGACTG	*Sma* I
Yeast one-hybrid and yeast two-hybrid	*MaMADS1-pGBKT7-F: AcatatgATGGGGAGGGGGAGGGTG*	*Nde* I
	MaMADS1-pGBKT7-R: AggatccTTCCAGCCATGCAGGCATAT	*Bam* H I
	MaNAC083-pGBKT7-F: AcatatgATGGATACGAAGCCGAGTGTTGTCGC	*Nde* I
	MaNAC083-pGBKT7-R: AggatccCTACGGAAGTGATGTGCCGGAACTGG	*Bam* H I
	MaACS1-promoter-pAbAi-F: AgagctcTAATCAAAATCAGACTGCATTGA	*Sac* I
	MaACS1-promoter-pAbAi-R: AgagctcGTGACCCGTTATCTCAGGTACG	*Sac* I
EMSA	*MaMADS1-pGEX-F: AgaattcATGGGGAGGGGGAGGGTG*	*Eco* R I
	MaMADS1-pGEX-R: ActcgagTTCCAGCCATGCAGGCATAT	*Xho* I
	MaNAC083-pGEX-F: AgaattcATGGATACGAAGCCGAGTGTTGTCGC	*Eco* R I
	MaNAC083-pGEX-R: ActcgagCTACGGAAGTGATGTGCCGGAACTGG	*Xho* I
	MaACS1-probe-F:TTCTTTCGCAAACAGCATGTCATCGATGAGATTAAG	
	MaACS1-probe-R:CTTAATCTCATCGATGACATGCTGTTTGCGAAAGAA	
	MaACS1-Mutant probe-F: TTCTTTCGCAAACAGCCCCCCCATCGATGAGATTAAG	
	MaACS1-Mutant probe-R: CTTAATCTCATCGATGGGGGGGCTGTTTGCGAAAGAA	
	MaACO1-probe-F: TGGAAGAACTCAACAACATGATGCCTTCCCGGGTCT	
	MaACO1-probe-R: AGACCCGGGAAGGCATCATGTTGTTGAGTTCTTCCA	
	MaACO1-Mutant probe-F: TGGAAGAACTCAACAGGGGGATGCCTTCCCGGGTCT	
	MaACO1-Mutant probe -R: AGACCCGGGAAGGCATCCCCCTGTTGAGTTCTTCCA	
	MaACO4-probe-F: TCACCCGAGCAGCGCCATGTGCAGATAAAAGAAACA	
	MaACO4-probe-R: TGTTTCTTTTATCTGCACATGGCGCTGCTCGGGTGA	
	MaACO4-Mutant probe-F: TCACCCGAGCAGCGCCCCCCGCAGATAAAAGAAACA	
	MaACO4-Mutant probe-R: TGTTTCTTTTATCTGCGGGGGGCGCTGCTCGGGTGA	
	MaACO5-probe-F: AACGTCCTCGAAGCTCACATGCGTTGCCGGGCTGAA	
	MaACO5-probe-R:TTCAGCCCGGCAACGCATGTGAGCTTCGAGGACGTT	
	MaACO5-Mutant probe-F:AACGTCCTCGAAGCTCGGGGGCGTTGCCGGGCTGAA	
	MaACO5-Mutant probe-R:TTCAGCCCGGCAACGCCCCCGAGCTTCGAGGACGTT	
	MaACO8-probe-F: TGTGAGACGGAGGAGCATGTTATGCATGACAACCGG	
	MaACO8-probe-R:CCGGTTGTCATGCATAACATGCTCCTCCGTCTCACA	
	MaACO8- Mutant probe-F: TGTGAGACGGAGGAGCCCCCTATGCATGACAACCGG	
	MaACO8-Mutant probe-R: CCGGTTGTCATGCATAGGGGGCTCCTCCGTCTCACA	
Subcellular localization	*MaMADS1-GFP-F: AggtaccATGGGGAGGGGGAGGGTG*	*Bam* H I
	MaMADS1-GFP-R: AaagcttTTCCAGCCATGCAGGCATAT	*Hind* III
	MaNAC083-GFP-F: AggtaccATGGATACGAAGCCGAGTGTTGTCGC	*Bam* H I
	MaNAC083-GFP-R: AaagcttCTACGGAAGTGATGTGCCGGAACTGG	*Hind* III
