# Supplementary material for: MaMADS1–MaNAC083 transcriptional regulatory cascade regulates ethylene biosynthesis during banana fruit ripening
Source: Hortic Res. 2023 Sep 8;10(10):uhad177. doi: 10.1093/hr/uhad177 (PMC10585711; doi:10.1093/hr/uhad177)
Supplement: Web_Material_uhad177 [file web_material_uhad177.zip › MaMADS1-MANAC083-Supplementary_Figures.doc]

**Supplementary Figures**

**Figure S1.** Comparison of ethylene biosynthesis genes expression between unripe and ripe banana fruit. (A) Photograph of unripe and ripe banana fruit. (B) Volcano plot comparing gene expression for ethylene biosynthesis genes in ripe fruit with that in unripe fruit as determined by transcriptome analysis. Red, green and gray dots represent up-regulated, down-regulated and non-significantly altered genes, respectively. (C) Heatmap of gene expression of *MaSAMS4*, *MaSAMS11*, *MaACS1*, *MaACS12*, *MaACO1*, *MaACO4*, *MaACO5*, and *MaACO8* in transcriptome analysis of unripe and ripe fruit. The green-black-red color and blue-white-purple scale represents log10 transformed FPKM value and log2 fold change, respectively.

**Figure S2.** The NAC recognition sequence (NACRS) in the nucleotide sequences of ethylene biosynthesis genes promoters. NACRS are indicated by red diamonds. The probe nucleotide sequence used in EMSA are listed below and the containing NACRS are identified in red font. The black vertical lines and numbers indicate the start and end positions of EMSA probes.

**Figure S3.** Recombinant purified GST-MaNAC083 was visualized on a Coomassie Brilliant Blue-stained SDS-PAGE. Lane 1, noninduced protein; Lane 2, before purifying the induced protein; Lane 3, purified induced protein.

**Figure S4.** The CArG box elements in the nucleotide sequences of ethylene biosynthesis genes promoters. CArG box elements are indicated by red diamonds. The probe nucleotide sequence used in EMSA are listed below and the containing CArG box elements are identified in red font. The black vertical lines and numbers indicate the start and end positions of EMSA probes.

**Figure S5.** Recombinant purified GST-MaMADS1 was visualized on a Coomassie Brilliant Blue-stained SDS-PAGE. Lane 1, noninduced protein; Lane 2, before purifying the induced protein; Lane 3, purified induced protein.

**Figure S6.** Molecular characteristics of MaMADS1. (A) Expression of *MaMADS1* in banana fruit during the ripening process. The relative expression level is shown as a ratio relative to that on day 0, which was set at 1. Data represented as means ± SE from three replicates, and distinct letters denote statistical variation (one-way ANOVA, *P* < 0.05) (B) *MaMADS1* promoter activity in response to ethylene. (C) Sub-cellular localization of MaMADS1 in tobacco BY-2 protoplasts. NLS-mCherry was used as a nuclear marker. Bars, 25 μm. (D) Transcriptional activation of MaMADS1 in yeast cells. The full length of MaMADS1 was fused with GAL4 DNA-binding domain. The pGBKT7 vector (BD) and p53-BD + T-antigen-AD were used as a negative and positive control, respectively. (E) Transcriptional activity analysis of MaMADS1 in tobacco leaves. The LUC/REN ratio of the empty pBD vector (negative control) was calibrated with 1. VP16 (an unusually potent transcriptional activator) was used as a positive control. Error bars in (B) and (E) denote S.E. from six replicates. Student’s *t*-test, ***P* < 0.01.
